# Successful implementation of an Enhanced Recovery After Surgery program shortens length of stay and improves postoperative pain, and bowel and bladder function after colorectal surgery

**DOI:** 10.1186/s12871-016-0223-0

**Published:** 2016-08-03

**Authors:** Ankit Sarin, Erik S. Litonius, Ramana Naidu, C. Spencer Yost, Madhulika G. Varma, Lee-lynn Chen

**Affiliations:** 1 Department of Anesthesia & Perioperative Medicine, University of California San Francisco, 505 Parnassus Ave. M917, San Francisco, CA 94143-0624 USA; 2 Department of Surgery, University of California San Francisco, 550 16th Street, 6th Floor, San Francisco, CA 94158 USA

**Keywords:** Enhanced recovery after colorectal surgery, Decreased length of stay, Outcomes

## Abstract

**Background:**

Despite international data indicating that Enhanced Recovery After Surgery (ERAS) programs, which combine evidence-based perioperative strategies, expedite recovery after surgery, few centers have successfully adopted this approach within the U.S. We describe the implementation and efficacy of an ERAS program for colorectal abdominal surgery in a tertiary teaching center in the U.S.

**Methods:**

We used a multi-modal and continuously evolving approach to implement an ERAS program among all patients undergoing colorectal abdominal surgery at a single hospital at the University of California, San Francisco. 279 patients who participated in the Enhanced Recovery after Surgery program were compared to 245 previous patients who underwent surgery prior to implementation of the program. Primary end points were length of stay and readmission rates. Secondary end points included postoperative pain scores, opioid consumption, postoperative nausea and vomiting, length of urinary catheterization, and time to first solid meal.

**Results:**

ERAS decreased both median total hospital length of stay (6.4 to 4.4 days) and post-procedure length of stay (6.0 to 4.1 days). 30-day all-cause readmission rates decreased from 21 to 9.4 %. Pain scores improved on postoperative day 0 (3.2 to 2.1) and day 1 (3.2 to 2.6) despite decreased opioid. Median time to first solid meal decreased from 4.7 to 2.7 days and duration of urinary catheterization decreased from 74 to 46 h. Similar improvements were observed in all other secondary end points.

**Conclusions:**

These results confirm that a multidisciplinary, iterative, team-based approach is associated with a reduction in hospital stay and an acceleration in recovery without increasing readmission rates.

**Electronic supplementary material:**

The online version of this article (doi:10.1186/s12871-016-0223-0) contains supplementary material, which is available to authorized users.

## Background

Significant improvements in recovery after major abdominal surgery can be achieved by implementing a standardized protocol of evidence-based treatments over the entire perioperative period [1–4]. First proposed by Kehlet et al. in 1994 [5, 6], this approach has coalesced into Enhanced Recovery After Surgery (ERAS) protocols which have evolved into a broader concept termed the Perioperative Surgical Home [7]. The underlying goals of this approach are to decrease variability in practice, reduce morbidity, enhance rate of recovery, and shorten postoperative length of stay (LOS).

Results from previously published reports [8–12] and systematic reviews [13–16] have been encouraging, with wide adoption in Europe [10]. However, in countries where healthcare management and resources are decentralized, ERAS programs can face substantial challenges for implementation. While there is a consistent body of literature validating the benefit of perioperative optimization, there are limited studies reporting successful implementation from the United States [17, 18].

We present a multi-disciplinary and iterative approach to implementing an ERAS protocol among patients undergoing abdominal colorectal surgery at University of California, San Francisco. During implementation, we monitored compliance on a monthly basis which directed us to areas for continuous process improvement in the protocol and led to targeted educational efforts for patients, faculty and staff. To evaluate the effectiveness of this program, we analyzed data on hospital LOS, readmission rates, pain management, and bowel and bladder function among 279 patients undergoing surgery after implementation of the ERAS program, and compared that with 245 consecutive patients who had undergone surgery immediately prior to implementation.

## Methods

The Institutional Review Board at the University of California – San Francisco (UCSF) approved the study in September 2013 (Study Number 13-11613). The study was conducted at one of the tertiary care teaching hospital sites of UCSF- Mount Zion Hospital. Four board-certified colorectal surgeons performed all surgeries.

### Multi-disciplinary team

We sought institutional commitment to develop an Enhanced Recovery After Surgery Program (ERAS) as a quality initiative and were given support of two personnel from the quality and safety program to assist with obtaining information on current quality and patient satisfaction. We were also given information technology support to develop a program for extracting data from our electronic medical record system. In July 2013, we created a multidisciplinary committee, ERAS Working Group, comprising of participants from Colorectal surgery, Anesthesiology, Pain management, Nursing, and Nutritional Services.

### ERAS phase

After reviewing published literature on successful ERAS programs, primarily from Europe, and following discussions with several national and international experts on ERAS, the Working Group developed a program for the perioperative management of patients having abdominal colorectal surgery at UCSF-Mt. Zion campus (See Additional file 1 - UCSF ERAS Protocol). The program provided a standardized pathway that guided the perioperative management of patients undergoing major abdominal colorectal surgery at UCSF-Mt. Zion. However, individual health care providers were allowed to deviate from the pathway if necessary. Preoperatively, patients were educated on the ERAS program and provided with written instructions and an informational pamphlet that also contained a brief exercise program with a focus on improving stability and mobility (See Additional file 2 - UCSF ERAS Patient Instructions). Patients were allowed to drink clear liquids until 4 h prior to the time of the procedure. Bowel preparation instructions did not change before and after the ERAS program. In general, surgeons avoided mechanical bowel preparation for right sided resections but gave a full bowel preparation for left sided and rectal lesions. Preoperatively, patients were given oral pre-emptive non-opioid analgesics, anti-emetic medications, deep vein thrombosis (DVT) prophylaxis and intravenous antibiotics. During surgery, guidelines for the conduct of anesthesia were provided, including restriction of intravenous fluids to approximately 2 l per routine case, use of epidural analgesia, minimizing opioid administration and providing adequate antiemetic prophylaxis. Postoperatively, the pathway provides specific instructions for non-opioid pain control, ambulation, DVT prophylaxis, and oral intake.

### Iterative evolution

Every month the ERAS Work Group met to discuss the implementation of the program. The group devised methods for capturing relevant data, analyzed the collected data, monitored protocol compliance and initiated changes to improve the outcomes for the patients. For example, we identified an area of potential low protocol compliance and attempted to verify its validity. Significant work was done to improve nursing reporting and documentation of parameters such as incentive spirometer use, ambulation and preoperative carbohydrate drink intake. This allowed us to improve our overall implementation.

### Study design

The program was introduced gradually in September 2013 and was fully in place by December 2013. We collected prospective demographic, co-morbidity, and perioperative data on consecutive patients undergoing abdominal colorectal surgery after implementation of the program from December 2013 to November 2014. We measured ERAS compliance by tracking the percentage of patients receiving epidurals and non-opioid medications, and percentage of patients ambulating daily.

The predetermined primary outcome measures were total hospital LOS, post procedure LOS and 30-day readmission rates. Total hospital LOS was defined as time from admission to discharge; the post-procedure LOS as the time from completion of surgery to discharge. Readmission was defined as any cause readmission to a system hospital within 30 days of surgery. Secondary outcome measures included postoperative pain scores, median opioid consumption intraoperatively and postoperatively in milligrams (mg) oral morphine equivalents, post-operative nausea and vomiting (PONV) rates, duration of epidural catheterization, duration of urinary catheterization, percentage of patients nil per os (NPO) on postoperative day (POD) 0 to POD 2 and number of days to first solid meal. Pain was assessed using the patient-reported Numerical Rating Scale 0 to 10, on which “0” represents no pain and “10” represents the worst possible pain.

After 1 year of data collection and protocol refinement (December 2013 to November 2014 – ERAS Phase), the number of patients (279) and procedures (310) were tabulated. We then reviewed the number of patients who had undergone major abdominal colorectal surgery in the months preceding the start of ERAS implementation. In order to achieve near-equivalent numbers of patients and procedures, we defined a Pre-ERAS phase as the 14 months from June 2012 to August 2013. This group was comprised of 245 patients having 298 procedures taken consecutively from operating room records. Retrospectively collected data from these Pre-ERAS phase patients were then compared to those patients undergoing surgery during the ERAS phase. We chose to exclude data collected between September and November 2013 during the rollout of the ERAS program to allow for adoption and training.

### Statistical analysis

Descriptive statistics of the pre-ERAS and ERAS groups were compared for all relevant patient characteristics, perioperative and postoperative data. Statistical comparisons between pre-ERAS and ERAS groups were performed using t-tests, Mann–Whitney U tests, and Fisher’s exact tests as appropriate. The statistical significance level for all comparisons was set at a two-tailed alpha = 0.05. All analyses were performed using R version 3.1.2 (R Foundation for Statistical Computing, Vienna, Austria).

## Results

There were 298 procedures on 245 individual patients in the Pre-ERAS group and 310 procedures on 279 individual patients in the ERAS Group. Relevant patient characteristics are shown in Table 1. There was no statistically significant difference in age, body mass index, or ASA rating. However, there were more men in the pre-ERAS group. Chronic health conditions such as diabetes and chronic cardiac conditions were equally represented in the two groups but rates of smoking and hypertension were higher in the pre-ERAS group than in the ERAS group. There were no patients or procedures excluded or deleted from either cohort of consecutive cases enrolled in the analysis.

**Table 1:** Patient Characteristics

	Parameter	Pre- ERAS group (Jun-12 to Aug-13)	ERAS Group (Dec-13 to Nov-14)	*p*
Procedures	Procedures excluding re-operations, *n*	298	310	
Individuals	Patients, *n*	245	279	
Age	Median age, years (Range)	54 (20–95)	53 (17–90)	0.44
Sex	Male patients, *n* (%)	169 (57 %)	137 (44 %)	0.0015
BMI	Median BMI, kg/m2 (Range)	25.5 (14.9–52.7)	24.1 (14.8–49.3)	
American Society of Anesthesiologists’ (ASA) classification	ASA Grade 1, *n* (%)	4 (1.3 %)	6 (1.9 %)	0.65
	ASA Grade 2, *n* (%)	199 (66.8 %)	208 (67.1 %)	
	ASA Grade 3, *n* (%)	90 (30.2 %)	95 (30.6 %)	
	ASA Grade 4, *n* (%)	5 (1.7 %)	1 (0.3 %)	
Smoker	Smokers, *n* (%)	24 (8.1 %)	9 (2.9 %)	0.0065
Diabetes	Diabetes, *n* (%)	23 (7.7 %)	21 (6.8 %)	0.75
Hypertension	Hypertension, *n* (%)	41 (14 %)	22 (7.1 %)	0.0077
CHD	Chronic heart disease, *n* (%)	25 (8.4 %)	14 (4.5 %)	0.068

Relevant surgical details are shown in Table 2. There was no significant difference in the primary indication for surgery. Colorectal cancer, inflammatory bowel disease, and other indications were equally represented in the pre-ERAS and ERAS groups. The number of patients who underwent open versus minimally invasive (laparoscopic / robotic) surgery in the pre-ERAS and ERAS groups was similar. The median duration of surgery was longer in the pre-ERAS group. The blood loss did not differ between the two groups. The distribution of types of cases between the two groups is shown in Table 3.

**Table 2:** Details of Surgery

	Parameter	Pre- ERAS group (Jun-12 to Aug-13)	ERAS Group (Dec-13 to Nov-14)	*p*
Indication for surgery	Colon or rectal cancer, *n* (%)	124 (42 %)	124 (40 %)	0.74
	Inflammatory bowel disease, *n* (%)	87 (29 %)	112 (36 %)	0.07
	Other indication for surgery, *n* (%)	94 (32 %)	87 (28 %)	0.38
Operative approach	Minimally invasive surgery, *n* (%)	155 (52 %)	184 (59 %)	0.073
	Planned Open surgery, *n* (%)	144 (48 %)	125 (40 %)	0.061
Length of procedure	Median Length of Procedure, Min (Range)	228 (30–1417)	196 (39–692)	0.01
Blood loss	Blood loss during surgery Median, ml (range)	100 ml (0–7800)	75 ml (0–6000)	0.58

**Table 3:** Case Mix – Top 10 Most Common cases

	Pre- ERAS group (Jun-12 to Aug-13)			ERAS Group (Dec-13 to Nov-14)		
	Procedure	*n*	Percent	Procedure	*n*	Percent
1	Takedown of loop ileostomy or colostomy, simple	35	11.7	Takedown of loop ileostomy or colostomy, simple	48	15.3
2	Laparoscopic low anterior resection with colostomy or loop ileostomy	23	7.7	Laparoscopic abdominal right colectomy	23	7.3
3	Laparoscopic sigmoid colectomy	20	6.7	Laparoscopic ileo-colectomy with ileocolic anastomosis	23	7.3
4	Laparoscopic abdominal right colectomy	18	6	Laparoscopic sigmoid colectomy	18	5.8
5	Abdominal perineal resection of rectum	15	5	Laparoscopic low anterior resection with colostomy or loop ileostomy	15	4.8
6	Completion proctectomy with creation of ileal reservoir and ileoanal anastomosis	13	4.3	Laparoscopic total abdominal colectomy with ileostomy	15	4.8
7	Laparoscopic total abdominal colectomy with ileostomy	13	4.3	Robotic assisted laparoscopic low anterior resection with anastomosis, colostomy or loop ileostomy	11	3.5
8	Laparoscopic ileo-colectomy with ileocolic anastomosis	12	4	Laparoscopic total proctocolectomy with ileoanal reservoir	10	3.2
9	Laparoscopic loop ileostomy	9	3	Takedown of colostomy from hartmann procedure	10	3.2
10	Abdominal exploratory laparotomy	8	2.7	Laparoscopic total proctocolectomy with ileostomy	9	2.9

Program adherence measures are shown in Table 4. A higher percentage of patients in the ERAS group received preoperative non-opioid analgesics (acetaminophen, diclofenac, gabapentin) as well as scopolamine. The percentage of patients receiving epidurals and transversus abdominis plane (TAP) blocks also increased significantly. The rates of ketamine infusion and ondansetron dosing intraoperatively did not differ significantly between the pre-ERAS and ERAS groups but the use of intraoperative dexamethasone was higher in the ERAS group.

**Table 4:** Program Adherence Measures

		Pre- ERAS group (Jun-12 to Aug-13)	ERAS Group (Dec-13 to Nov-14)	*p*
Pre-operative non opioid medications usage	Acetaminophen *n* (%)	28 (9.4 %)	277 (89.4 %)	<0.001
	Diclofenac *n* (%)	2 (0.7 %)	104 (33.5 %)	<0.001
	Gabapentin *n* (%)	20 (6.7 %)	281 (90.6 %)	<0.001
	Scopolamine *n* (%)	2 (0.7 %)	68 (21.9 %)	<0.001
Regional analgesia usage	Epidural used for intraoperative analgesia, *n* (%	78 (26.2 %)	185 (59.7 %)	<0.001
	Epidural used for postoperative analgesia, *n* (%)	86 (29 %)	210 (68 %)	<0.001
	TAP block given for intraoperative analgesia, *n* (%)	9 (3 %)	26 (8.4 %)	0.005
Adjunctive intraoperative medications	Intraoperative Ketamine Infusion, *n* (%)	20 (6.7 %)	29 (9.4 %)	0.24
	Intraoperative Dexamethasone, *n* (%)	90 (30.2 %)	133 (42.9 %)	0.0014
	Intraoperative Ondansetron, *n* (%)	260 (87.2 %)	272 (87.7 %)	0.9
Chemical DVT prophylaxis	Preoperative DVT prophylaxis with subcutaneous Heparin, *n* (%)	100 (34 %)	273 (88 %)	<0.001
Preoperative antibiotic	Intravenous antibiotics given before incision, *n* (%)	268 (90 %)	300 (97 %)	0.0008
Intraoperative fluids	Median introperative crystalloid, ml (min, 1st Q, 3rd Q, max)	1350 (0, 800, 2000, 30,000)	1500 (0, 862.5, 2500, 18,000)	0.027
	Median intraoperative colloid, ml (min, 1st Q, 3rd Q, max)	0 (0, 0, 500, 6000)	0 (0, 0, 0, 4500)	<0.001
Post-operative non opioid medications scheduled usage	Acetaminophen *n* (%)	192 (64.4 %)	289 (93.2 %)	<0.001
	Diclofenac *n* (%)	5 (1.7 %)	70 (22.6 %)	<0.001
	Gabapentin *n* (%)	23 (7.7 %)	254 (81.9 %)	<0.001
	Scopolamine *n* (%)	80 (26.8 %)	188 (60.6 %)	<0.001
Adherence to ambulation	Patients Ambulating on POD 0, *n* (%)	15 (5.0 %)	309 (99.7 %)	<0.001
	Patients Ambulating on POD 1, *n* (%)	91 (30.5 %)	307 (99.0 %)	<0.001
	Patients Ambulating on POD 2, *n* (%)	97 (32.6 %)	299 (96.5 %)	<0.001

As shown in Table 4, the rates of intraoperative chemical DVT prophylaxis and IV antibiotic use prior to the procedure also increased. We found that the median volume of crystalloid used was lower in the pre-ERAS than in the ERAS group. However, the median volume of colloid used was significantly lower in the ERAS group. Postoperatively, a significantly higher percentage of patients in the ERAS group received scheduled non-opioid analgesics (acetaminophen, diclofenac, gabapentin) as well as scopolamine. Percentage of patients ambulating on POD 0 to 2 was also significantly higher in the ERAS group compared to the pre-ERAS group.

Table 5 shows the results of the primary outcome measures. As shown, there was a reduction in the median total hospital LOS (from 6.4 to 4.4 days) as well as a reduction in post procedure LOS (from 6.0 days to 4.1). In addition, the 30-day readmission rate was significantly lower for the ERAS group at 9.4 % compared to 21 % for the pre-ERAS group.

**Table 5:** Primary Outcome Measures

Parameter	Pre- ERAS group (Jun-12 to Aug-13)	ERAS Group (Dec-13 to Nov-14)	*p*
Median total hospital length of stay from admission to discharge, days (range)	6.4 (0.2–197.7)	4.4 (1.0–80.4)	<0.001
Median post procedure length of stay from end of procedure to discharge, days (range)	6.0 (0.1–161.5)	4.1 (0.8–47.0)	<0.001
Readmission rate 30 day all cause readmission rate, *n* (%)	64 (21 %)	29 (9.4 %)	<0.001
Reoperation rate reoperation for any indication within 30 days, *n* (%)	5 (2 %)	6 (2.1 %)	1

Outcomes of the pain management protocol are shown in Table 6. The median opioid consumption (in mg oral morphine equivalents) decreased in the ERAS group compared to the pre-ERAS group both intraoperatively and postoperatively. The median duration of epidural catheterization also decreased significantly from 95 to 52 h. Patient self-reported postoperative pain scores were significantly improved on POD 0 and 1 but no difference was found in the two groups on POD 2.

**Table 6:** Secondary Outcome Measures - Pain management

		Pre- ERAS group (Jun-12 to Aug-13)	ERAS Group (Dec-13 to Nov-14)	*p*
Median opioid consumption Intraoperative (mg po morphine equivalents)	Intraoperative opioid consumption, mg (range)	99.0 (0.0–605.0)	68.0 (0.0–293.0)	<0.001
Median opioid consumption from POD 0 to POD 2 (mg po morphine equivalents)	IV and PO opioid consumption, mg (range)	142.2 (0.0–1964.0)	75.0 (0.0–3162.0)	<0.001
	Epidural opioid consumption, mg (range)	299.3 (7.6–1017.1)	209.8 (7.8–788.5)	<0.001
Median Duration of Epidural catheterization (in hours)	Epidural duration – median, H (range)	95 (25–264)	52 (3–261)	<0.001
Median Patient self-reported pain scores from 10 (worst) to 0 (no pain).	POD 0 score, *n* (range)	3.2 (0.0–8.8)	2.1 (0.0–9.3)	<0.001
	POD 1 score, *n* (range)	3.2 (0.0–8.3)	2.6 (0.0–9.6)	0.0019
	POD 2 score, *n* (range)	2.5 (0.0–10.0)	2.7 (0.0–9.1)	0.77

Effect of the program on bowel and bladder function is shown in Table 7. PONV was measured as a combination of antiemetic use and patient self-reported nausea or vomiting in the immediate postoperative period. While there was no difference in the amount of anti-emetics used in the pre- ERAS and ERAS groups, the subjective reporting of nausea/vomiting by patients was significantly reduced in the ERAS group. We used sequential dietary orders as a surrogate for bowel function. In comparing the pre-ERAS group to the ERAS group, the percentage of patients who were NPO decreased from 61.7 to 29 % on POD 0; 30.9 to 14.5 % on POD1 and 12.1 to 9.4 % on POD 2 respectively. While the difference in POD 0 and 1 was statistically different, it was not on POD 2. The median time to first solid meal decreased significantly from 4.7 days in the pre-ERAS group to 2.7 days the ERAS group. The median duration for urinary catheterization decreased from 74 h in the pre-ERAS group to 46 h in the ERAS group.

**Table 7:** Secondary Outcome Measures - Bowel and Bladder function

		Pre- ERAS group (Jun-12 to Aug-13)	ERAS Group (Dec-13 to Nov-14)	*p*
Need for postoperative antiemetics	Received antiemetic in post anesthesia care unit, *n* (%)	181 (60.7 %)	207 (66.8 %)	0.13
Patient self reported postoperative nausea and vomiting	Postoperative nausea and vomiting in post anesthesia care unit, *n* (%)	125 (41.9 %)	74 (23.9 %)	<0.001
Percentage of patient ordered nothing by mouth (NPO) post-operatively	Patients NPO on POD 0, *n* (%)	184 (61.7 %)	90 (29.0 %)	<0.001
	Patients NPO on POD 1, *n* (%)	92 (30.9 %)	45 (14.5 %)	<0.001
	Patients NPO on POD 2, *n* (%)	36 (12.1 %)	29 (9.4 %)	0.13
Days from admission to 1st solid meal	Median time to first solid diet, days (range)	4.7 (0.6–23.7)	2.7 (0.8–37.7)	<0.001
Duration of urinary catheterization with a Foley Catheter (in hours)	Foley Duration –Median, h (range)	74 (2–649)	46 (1–2262)	<0.001

## Discussion

Our development of a multidisciplinary, evidence-based, enhanced recovery after surgery program at a major tertiary medical center performing abdominal colorectal surgery was associated with shortened LOS and reduced readmission rates. We were also able to show an improvement in pain control, and reduced opioid consumption and postoperative nausea – all leading to an accelerated return of bowel and bladder function. These results are consistent with findings from other centers and in varied patient populations [8, 11–20] and lends impetus to the push for further incorporation of such programs in current clinical practice within in the U.S.

Patient education and defining expectations were cornerstones of our program to ensure patient participation is well-established [21, 22]. Comprehensive patient instructions including a website (http://eras.surgery.ucsf.edu) allowed for a step-by-step overview of the program. Patients who were informed to walk on the day of surgery and to be home in 3–4 days were more likely to accept this newer paradigm. The preoperative exercise program in the patient instructions was designed in collaboration with a physical therapist. It focused on improving balance and core strength to facilitate stability and mobility after the operation. Similarly, incentive spirometer teaching occurred preoperatively, which established baseline spirometer goals for patients to work towards postoperatively.

There has been a major move away from prolonged fasting to permitting clear fluids up to two hours prior to surgery and solids up to 6 h prior to surgery in accordance with the ASA guidelines. In keeping with the evidence [23–26], our patients were allowed clear liquids and asked to drink a carbohydrate-rich clear liquid up to 4 h prior to surgery. Four hours was chosen as a cut off to allow for flexibility in the event the procedure could be done earlier than anticipated. Benefits of the nutritional drink include reduced catabolism, improved postoperative insulin sensitivity, reduced LOS and improved patient satisfaction by reducing preoperative thirst, hunger, and discomfort [23, 27–31].

Even though the carbohydrate drink requirement was a part of the protocol and not optional, rates of adherence to this component were quite variable. Our analysis of the reasons for this variability included (1) availability of the drink to give to patients in the clinic, (2) whether the surgery was booked in the clinic vs. over the phone and (3) patient concerns with violating the traditional “NPO after midnight” instruction. In addition, we were hampered by uneven charting of NPO status and carbohydrate intake by preoperative nursing personnel. Over time, the rates of adherence did improve due to the focused efforts of the Working Group to improve compliance with this element of the pathway.

Intraoperatively, fluid restriction was an established practice prior to the program based on studies showing reduced complications [32, 33]. The goal was to maintain a fluid restrictive strategy that was statistically significant; however, a small (150 ml) increase in crystalloid administration was detected. Despite some recent evidence suggesting that fluid restriction may not accelerate recovery as previously thought [34, 35], we believe that our findings reflect a trend towards use of crystalloid in preference to colloid administration. Additionally, we increased the compliance of perioperative DVT prophylaxis and antibiotic administration [36] quality, perhaps as a result of increased attention.

Optimizing perioperative pain management while reducing opioid use were major goals of the ERAS program. Our pain management strategy incorporated recent evidence and expert opinion in designing the analgesic protocols [37] with modifications in the use of neuraxial anesthesia, ketamine, acetaminophen, gabapentin and COX-inhibitors.

We chose to use thoracic epidural analgesia (TEA) despite recent literature [37, 38] suggesting that recovery from laparoscopic colorectal procedures may not benefit from TEA. However as a tertiary referral center, redo operations, pelvic surgeries and stomas are more commonplace than simple colectomies. The routine use of a hand-assisted port, typically involving a 6–8 cm infra-umbilical vertical or transverse incision and a significant number of ‘open’ surgeries suggested important benefit would be derived from placement of TEA. [39] We chose to use a low concentration of ropivacaine (0.0625 %) to mitigate risk of hypotension, motor dysfunction, and ease the transition when the epidural is removed on POD 2. In addition, all patients with TEA were followed by an inpatient acute pain service that adjusted the TEA infusion rates to optimize pain control and prevent lower extremity motor weakness, which would have impaired early mobilization.

Patients, who required more than 100 mg oral morphine equivalents per day, had a sub-anesthetic dose of ketamine infusion added in their therapy for anti-hyperalgesia. Additional benefits included the reduction in opioid consumption by 35–50 % [40, 41] and a decreased development of persistent post-surgical pain [42, 43] without significant adverse effects [43]. Other opioid-sparing strategies included administration of acetaminophen and gabapentin through all phases of care.

Adjuncts used for PONV such as dexamethasone and scopolamine may have also contributed to post-operative pain management and opioid reduction [44, 45]. There were improvements in early postoperative pain scores as well as decreased overall opioid consumption when we followed an evidence-based multimodal pain management strategy.

Early mobilization and diet advancement have become a foundation of any rapid recovery protocol [46, 47]. We instituted a similar protocol and combined these measures with rapid de-escalation of fluids and gum chewing, which have also been shown to hasten bowel recovery [48, 49].

The pre-ERAS and ERAS groups are well-balanced with no difference in age, BMI, morbidities (ASA classification), indication for surgery as well as operative approach (open versus minimally invasive).

We chose LOS as our primary outcome measure as a surrogate of postoperative inpatient recovery. Rapid recovery from surgery and therefore early discharge is limited by pain, organ dysfunction, nausea and vomiting, ileus, hypoxemia, fatigue and immobilization [50]. In addition, LOS is also influenced by demand factors (patient’s need for care influenced by age, severity of disease and complications, co-morbidity and social circumstances [51] and supply factors including clinical practice style, availability of beds and discharge policies [52, 53]. The goal of building a multifocal accelerated pathway is to influence all of these factors to some degree. Therefore, LOS serves as a reasonable measure to assess the efficacy of this type of intervention.

Total LOS and post-procedure LOS were assessed independently. For example, patients with inflammation or obstruction were often medically treated before surgical intervention. Readmission rates were also a primary outcome measure. Adopting an early discharge strategy that substituted additional days spent in the hospital with a higher readmission rate would only shift recovery to home rather than enhancing recovery. As noted, we were able to show that an ERAS program reduced readmission rates while shortening LOS.

Recovery of bowel function was assessed using immediate PONV and time to first meal. We tracked both anti-emetic use and patient reporting of nausea. We were unable to detect a change in the amount of anti-emetics administered postoperatively; however, patients reported feeling better in the immediate postoperative period. This finding may be directly related to application of preoperative scopolamine patch along with intraoperative administration of dexamethasone and ondansetron. Reduced opioid consumption may also be a contributing factor to reduced nausea. We were able to show a significant improvement in bowel recovery when measured by progressive percentage of patients NPO postoperatively and time to first solid meal. As expected by POD 2, the two groups were equivalent. We did not track time to first flatus or first bowel movements as we feel these measures are less reliable. Diet advancement was typically a function of patient appetite and this has been shown to be good indicators for bowel function [54].

Return of bladder function was measured in terms of duration of urinary catheterization, which was significantly less in the ERAS group. We did attempt to determine catheter re-insertion rates but reporting and tracking was poor as a wide variety of criteria were used to reinsert catheters or to use a straight catheter for one time assistance with voiding based on individual provider preference.

It remains to be seen if ERAS programs improve patient satisfaction while accelerating recovery [55]. In the future, we intend to advance the perioperative surgical home model by investigating patient satisfaction and financial impacts of ERAS programs compared to traditional care. We believe these studies are important to help guide healthcare in the direction of better quality and better value.

### Limitations

Retrospectively analyzing data from a prospective program especially one directed towards continuous quality improvement comes with its own challenges. Although the two groups were fairly well balanced, there were some differences. Of note, the pre-ERAS and ERAS groups had significantly different rates of smoking and hypertension. Our only explanation for the statistically significant differences in rates of smoking and hypertension between the two groups is that the ERAS group was temporally after the pre-ERAS group and the difference may be a reflection of the medical center’s continued push to decrease tobacco utilization amongst patient and in the community. Such differences are to be expected in a retrospective analysis without randomization. As stated above, no patients were excluded from either cohort to reach complete balance of these associated medical conditions.

Given the evidence-based nature of the ERAS program, it would have been unethical to withhold such care in the control arm of a randomized clinical trial. We were also resource-constrained in finding matching controls in historical data, since we switched to a new electronic medical system in June 2012 from when data was systematically available. Due to the wide area serviced by a tertiary care center, we are unlikely to have captured all readmissions if patient were readmitted to local hospitals outside of our system. This rate should not be different for our two study groups; therefore, we are confident in our finding of reduced readmission rates. Specific complication rates were not tracked. Reduced LOS and reduced readmission rates served as surrogate markers of reduced complication rates since any significant complications would have adversely affect one or both of those outcome measures. The 30-day mortality rate for both groups was zero and the re-operation rate was not different. Although we have shown a significant improvement in various outcomes, we were unable to determine the degree of impact of the various components of the program and separate the critical components from the ones that were adjuncts. This is an area of future focus.

## Conclusion

Use of a multidisciplinary evidence-based program is associated with improved outcomes and decreased recovery time in the perioperative care of patients undergoing abdominal colorectal surgery. While numerous models for such programs are available for review, a successful program requires input from several key disciplines and adaptation to local factors. Adoption of such programs in the care of surgical patients should be encouraged.

## Abbreviations

DVT, deep vein thrombosis; ERAS, enhanced recovery after surgery; LOS, length of stay; mg, milligrams; NPO, nil per os; POD, post operative day; PONV, post-operative nausea and vomiting; UCSF, University of California at San Francisco

## Additional files

Additional file 1: UCSF ERAS Protocol. (PDF 35 kb)

Additional file 2: UCSF ERAS Patient Instructions. (PDF 153 kb)
