# Supplementary material for: Successful implementation of an Enhanced Recovery After Surgery program shortens length of stay and improves postoperative pain, and bowel and bladder function after colorectal surgery
Source: BMC Anesthesiol. 2016 Aug 3;16:55. doi: 10.1186/s12871-016-0223-0 (PMC4973042; doi:10.1186/s12871-016-0223-0)
Supplement: Supplementary file 1 — UCSF ERAS Protocol. (PDF 35 kb) [file 12871_2016_223_MOESM1_ESM.pdf]

Phase	Section	Subsection	ANESTHESIA		SURGERY	NURSING	PATIENT
DAYS B4	PREPARE		Phone Consult or Appointment		Enter pre-op orders		Enroll in MyChart
			Deliver instructions via MyChart or mail.		Patient Education in Clinic		Prehabiliation: Follow Exercise program in patient instructions
					Stoma marking if applicable		Visit ERAS website for information
DOS. PRE-OP			Pre-Op Warming. PIV. Crystalloid @ 30 ml/hr		No bowel prep for ileocolic, right, transverse, colectomy	Please complete Pre-Op RN checklist 45 minutes prior to OR start time, then Green Light.	Nothing by mouth for eight hours before surgery except for a Boost Breeze completed 2 hours before coming to hospital.
	MEDICATIONS	ANALGESICS	Gabapentin	600mg once	Nothing by mouth for eight hours before surgery except for a Boost Breeze completed 2 hours before coming to hospital.		
			Acetaminophen	1000mg once		Apply Warming Blanket to patient. Teach IS.	Risks of surgery and anesthesia will be discussed. You will sign a consent for the procedure, and discuss the possibility of receiving blood products.
			Diclofenac (if eGFR>60)	100mg once		IV Placed. Crystalloid started at 30ml/hr.	
		PONV	Scopolamine	1.5mg TD once		Gabapentin 600, APAP 1000, Diclofenac given once with water (<100ml). Antiemetics may also be ordered.	
			Age < 60 years		Consent checked, Site Marking, and 24-hr H&P completed 40 minutes before OR start time.		If there is any chance you might be pregnant, please discuss with surgery and anesthesia
	REGIONAL		30 minutes before start time, complete anesthesia assessment, go to Block Room, and place Thoracic Epidural placed at T8-10				
INTRA-OP	SCIP-VTE		Heparin 5000units SQ X 1 after epidural placement				
			Orogastric tube to low intermittent suction.		If on steroids, ask for Hydrocortisone 100mg IV x 1		
			Fluids: NTE 2L unless EBL>300ml,				
	SCIP-10		Patient temperature must not drop below 36.0 C.				
	SCIP-Inf-1,2		Antibiotic: Ertapenem	1 gram IV x 1			
	MEDS		Opioid of Choice: Hydomorphone or Morphine. Titrate to RR 12bpm at time of extubation.				
			If Opioid-Tolerant, continue their opioid regimen intra-op. Start ketamine load and infusion. 0.2 mg/kg x 1. Then 2 mcg/kg/min.				
		PONV	Dexamethasone	4mg IV x 1 after induction			
			Metoclopramide 10mg IV X 1. Unless contraindicated.				
			Ondansetron	4mg IV x 1			
	REGIONAL	O R	Thoracic Epidural 0.0625% Ropi + Fentanyl 2 mcg/ml @ 8 ml/hr				
			Bilateral TAP Block. 20ml of Ropi 0.2%. Done prior to prep & drape.				
PACU	MEDICATION		Order opioid of Choice: Hydomorphone or Morphine. May start PCA.		Order Postop Colorectal Surgery Orderset	Hydomorphone or Morphine IV PRN Titrate to RR 10bpm	
			Order Antiemetics		Choose Famotidine (if GERD or steroids)	Thoracic Epidural 0.0625% Ropi + Fentanyl 2 mcg/ml @ 8 ml/hr	
	REGIONAL		Thoracic Epidural 0.0625% Ropi + Fentanyl 2 mcg/ml @ 8 ml/hr		Choose Toradol if appopriate		

**FLOOR/ICU POD 0**	**MEDICATIONS**	Gabapentin	600mg PO QHS		Vital Signs q 4H, I&O shift, weight daily, surgical incision care abdomen,	Out of bed 6 hours after surgery
		Acetaminophen	1000mg IV q6H		Out of bed 6 hours after surgery with assistance of Nursing	Incentive Spirometry x10 q 1H
		Toradol (if eGFR>60)	15mg IV q6H		Encourage Incentive Spirometry x10 q 1H	Limited Clears (<500 ml per shift)
		If Opioid-Tolerant, continue ketamine infusion 2 mcg/kg/min and maintain daily opioid req.			Foley Catheter to gravity.	Gum Chewing encouraged
		PCA HM 0.2/10/0 or Oxycodone PRN			DVT Proph: Heparin 5kU SQ TID	
	**REGIONAL**	Thoracic Epidural 0.0625% Ropi + Fentanyl 2 mcg/ml @ 8 ml/hr				
**FLOOR/ICU POD 1**	**MEDICATIONS**	Gabapentin	600mg PO QHS	Evaluate IV Fluids and avoid hypervolumia	Vital Signs q 4H, I&O shift, weight daily, surgical incision care abdomen,	Walking 5 times a day. At least first time with nurse.
		Acetaminophen	1000mg IV/PO q6H	Labs: CBC, Cr, BUN	Ambulation: OOB to chair (3hrs) BID Ambulation 5 x per day	Incentive Spirometry x10 q 1H
		Toradol OR Diclofenac (eGFR)	15mg IV q6H 50mg PO TID	Advance diet to unlimited clears if tolerated and reg food later if doing well	Encourage Incentive Spirometry x10 q 1H	Unlimited Clears or Low residue diet
		If Opioid-Tolerant, continue ketamine infusion 2 mcg/kg/min and maintain daily opioid req.		Address Foley removal either today o POD 4 (if pelvic dissection)	Remove Foley Catheter in AM	Gum Chewing OK
		PCA HM 0.2/10/0 or Oxycodone PRN		Address steroid taper if appropriate	DVT Proph: Heparin 5000U SQ TID	
	**REGIONAL**	Thoracic Epidural 0.0625% Ropi + Fentanyl 2 mcg/ml @ 8 ml/hr			Unlimited clears or soft Diet. Gum chewing ok.	
**FLOOR/ICU POD 2**	**MEDICATIONS**	Gabapentin	600mg PO QHS	Evaluate IV Fluids and avoid hypervolumia	Vital Signs q 4H, I&O shift, weight daily, surgical incision care abdomen,	Walking 5 times a day.
		Acetaminophen	1000mg IV/PO q6H	Labs: CBC, Cr, BUN	Ambulation: OOB to chair (3hrs) BID Ambulation 5 x per day	Incentive Spirometry x15 q 1H
		Toradol OR Diclofenac (eGFR)	15mg IV q6H 50mg PO TID	Regular diet /Low residue for new ileostomies or newly working ileoanals	Encourage Incentive Spirometry x10 q 1H	Regular diet / Low residue diet
		If Opioid-Tolerant, continue ketamine infusion 2 mcg/kg/min		Nutrition Consultation for new ileostomies or new working ileoanal anastomosis		
		If Opioid-Tolerant, continue their daily opioid requirement.		Hold 6AM Heparin dose	DVT Proph: Heparin 5000U SQ TID	
	**REGIONAL**	STOP epidural infusion at 6AM. Catheter to be removed at 8AM.		Address steroid taper if appropriate	Regular diet / Low residue diet	
