# Supplementary material for: Successful implementation of an Enhanced Recovery After Surgery program shortens length of stay and improves postoperative pain, and bowel and bladder function after colorectal surgery
Source: BMC Anesthesiol. 2016 Aug 3;16:55. doi: 10.1186/s12871-016-0223-0 (PMC4973042; doi:10.1186/s12871-016-0223-0)
Supplement: Supplementary file 2 — UCSF ERAS Patient Instructions. (PDF 153 kb) [file 12871_2016_223_MOESM2_ESM.pdf]

ENHANCED RECOVERY AFTER SURGERY

Your Surgeon has determined that you a good candidate for our Enhanced Recovery after Surgery (ERAS) Program. This program uses the best practices in surgical care. It will help you recover and get home as quickly and as safely as possible after your surgery. Please read this handout about ERAS to get the most out of the program.

OUR APPROACH TO RECOVERY AFTER COLON & RECTAL SURGERY

Your surgical team will:

- Use the most modern anesthesia methods
- Promote return of bowel function as quickly as possible
- Use best practices for diet, bowel preparation, antibiotics and hydration.
- Encourage YOU to ambulate early after surgery
- Provide alternatives to narcotics to effectively treat pain.
- Help you start drinking and eating soon after surgery with easy-to-digest liquids and foods

WHY IS THIS IMPORTANT?

- Simple things matter !!
- **Common, yet serious, complications can help be prevented by getting out of bed, sitting in chair and walking**
- YOU CAN HELP AVOID:
  - Pneumonia
  - Blood clots
  - Bed sores
  - Prolonged “sleeping bowels” or ileus

BEFORE YOUR SURGERY - AT HOME PRIOR TO SURGERY

- Continue regular exercise up until the date of your surgery. See below.
- If you smoke, quit today. Quitting smoking is the best way to avoid breathing problems! Quitting smoking will help you heal!
- **UCSF MyChart** is computer program that is an easy, confidential way to stay in touch with UCSF Medical Center. This is a secure online resource to communicate with your providers. Sign-up for it at https://ucsfmychart.ucsfmedicalcenter.org
- A week before surgery you will receive a phone call from the PREPARE clinic to set up either a phone consult or in-person pre-operative clinic appointment. The number for the PREPARE clinic is 415-XXX-XXXX in case you need to schedule a visit.
- Purchase Boost Breeze or Ensure Clear (a nutritional drink) as you have it to drink before you come to the hospital. (More details below).
- Purchase sugarless gum or hard candy for use after your surgery.
- Arrange to have someone take you home after you are discharged. This may be two to three days after surgery.

Exercises before surgery that can aid Recovery

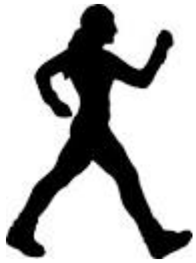

Walking:

At least 20 minutes, 5 times/week

This should be in addition to normal activity

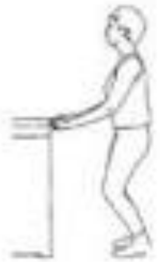

Mini squats

Stand at counter, using fingers for balance as needed

Place feet hip width apart

Sit down and back as if you are sitting in a chair

Do not let your hips drop below your knees

Repeat 10 times, 2 times/day

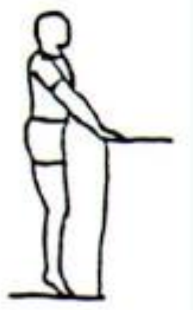

Heel raises

Stand at a counter, use fingers for balance as needed

Raise up on your toes, lifting heels off the ground

Stay for 1-2 seconds and SLOWLY lower your heels

Repeat 15 times, 2 times/day

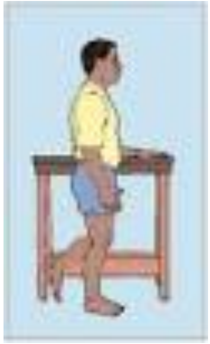

Single leg stance

Stand at counter, using fingers for balance as needed

Lift LEFT leg and hold for 30 seconds

Lower and repeat with RIGHT leg

If that gets too easy, try to hold your balance without using your hands for balance, or close your eyes

Perform 2 times/day for 30 seconds on each leg

THE DAY BEFORE SURGERY

- The day before the surgery: you will do one of the following as already instructed:
    - Do a Bowel prep with Golytely or equivalent
    - Do a Bowel prep with Fleet's enema
    - Not need to do any bowel prep
- If you have any questions about this, call your surgeon's office as soon as possible.
- Shower the evening before or morning of surgery with either **Dial antibacterial or chlorhexidine soap** (Hibiclens). Wash your entire body. Do not use chlorhexidine (Hibiclens) on your face because it can damage your eyes. These soaps are available at most drug stores (for example Walgreens, Target, Wal-Mart, CVS, or Rite Aid).
- The day before your surgery you should not have any solid food and should only have clear liquids.
- You can drink clear liquids up until **2 hours before you arrive at the hospital.** Clear Liquids are limited to:
    - ♣ Clear Broth
    - ♣ Clear juices such as apple, cranberry and grape. (NO orange juice)
    - ♣ Jell-O
    - ♣ Popsicles
    - ♣ Coffee/tea (no milk or cream)
- Wear comfortable clothing and leave all valuables at home.
- Wear your eyeglasses and bring a case. (no contact lenses)
- If you get sick before surgery (fever, cough, sore throat, cold, flu, infection), please call your surgeon and the Mount Zion Adult Prepare Clinic at 415-885-7670.
- You may bring toiletries if you desire; otherwise the hospital will provide them for you.
- **DO NOT** bring your medications with you to the hospital unless you were specifically instructed to do so.
- **BRING** a list of your medications including dose(s) and when you take them.
- **BRING TWO forms of ID** - including one ID with a photo.

ON THE DAY OF SURGERY

- Come to Mount Zion Hospital 1600 Divisadero. Check in at Admitting, Room #A108 at assigned ARRIVAL time (two hours before the scheduled surgery). Admitting will direct you to the Pre-Op Nursing Station on the third floor.. Phone # (415) XXX-XXXX
- Drink 1 container (8 ounces) Boost Breeze or Ensure Clear **2 HOURS BEFORE ARRIVAL** at hospital.
    - - For example, if arrival time at hospital is 6am, drink the Boost Breeze at 4am.
      - If you have Diabetes, drink only half the container (4 ounces).
      - Boost Breeze is available at the UCSF Mount Zion Gift shop. It comes in 8 ounce containers and costs $2.50 each in all flavors; wild berry, orange and peach.
      - This special drink provides your body with energy during the operation.
- If you have been instructed to take any medications the morning of surgery, take them with a small sip of water.
- Nothing more to drink after the Boost Breeze / Ensure Clear

- ***If you eat or drink anything else after that time your surgery may be cancelled or delayed***

What to expect when you come in for surgery:

- You will get IV fluids
- A warming blanket will keep you warm to help prevent infection after surgery.
- You will learn to use incentive Spirometer, a breathing exerciser. This can help prevent pneumonia. It is important to use it on your own to help with your recovery.
- You will sign a consent form for surgery and the possibility of a blood transfusion.
- If you think you might be pregnant tell your surgeon or nurse.
- You may have an epidural placed before the surgery to reduce postoperative pain.
- You may have an anti-nausea patch placed behind your ear.
- You will receive two or three pain medications to help with pain control. These pills include:
  - - Gabapentin: Blocks nerve related pain
  - - Acetaminophen (Tylenol)
  - - Diclofenac: Anti-inflammatory pain medicine

AFTER YOUR SURGERY - ON THE DAY OF SURGERY (Post-Op Day 0)

- You will get out of bed with assistance on the day of surgery itself.
  - Getting out of bed can reduce complications.
  - Staying active is the most important thing you can do to facilitate your recovery
- You will most likely have a PCA (Patient controlled analgesia) pump to help with pain control. Your nurse will show you how to use it.
- A urinary catheter (which was put in while you were asleep) will be removed the day after surgery.
- Unless instructed differently, it is OK for you to take clear liquids and chew gum or hard candy on the day of your surgery.
  - ♣ Clear Broth
  - ♣ Clear juices such as apple, cranberry and grape.
  - ♣ Jell-O
  - ♣ Popsicles
  - ♣ Coffee/tea (no milk/cream)
- You may drink liquids as you like, but do not push yourself to drink. If you become nauseated, stop drinking and tell your nurse.

THE DAY AFTER SURGERY (Post-Op Day 1)

- GET UP AND GET MOVING! This is **extremely important** after surgery.
- Plan on walking at least five times a day and sit in a chair for at least six hours.
  - The first time you walk will be with your nurse. Your nurse will tell you when you are safe to walk by yourself.
  - Getting out of bed, sitting and walking will help with breathing, circulation, your bowels and your wellbeing. It may even REDUCE PAIN!
  - Activity will not damage the surgical area.

- **Staying active is the most important thing you can do to help your recovery and prevent complications**

- Use the incentive spirometer 10 times every hour you are awake.
- The urinary catheter will be removed in the morning. **Remind your care team if you have not been given a reason and you still have it in at noon.**
- Unless your surgeon tells you differently, it is OK for you to take soft foods the day after your surgery.
  - At first, small frequent meals may be best after abdominal surgery. Don't force yourself to eat.
  - - Tell your nurse if you have nausea.
- Once you are drinking liquids, you will be given pain pills. . The pills work best when used regularly, so ask for another dose before the pain gets too bad.
- We try to keep you comfortable after your surgery, but expect some discomfort as you move about. We try to balance minimizing pain and avoiding the side effects of the pain medication. **Expect soreness after surgery but let you team know if you need more help to control your pain.**
- Help your nurse record how much you eat and drink and how much you urinate.
- **You will be given an activity journal. Fill it out with pride!**

TWO DAYS AFTER SURGERY (Post-Op Day 2)

- Your epidural catheter will probably be removed this morning. Pain should be well controlled with pain pills. Soreness is to be expected but it should not limit your ability to be active.
- Write down questions to discuss with your team. Your nurse will go over instructions with you.
- Continue to walk at least five times daily and have a family member help you if you need it.
- Remember. Even when you are at home you are never more than a phone call away from your care providers at UCSF.

AT HOME AFTER DISCHARGE

- Make an appointment to see your surgeon 2-4 weeks after surgery. Call 415-XXX-XXX to make an appointment, or schedule through MyChart.
- Call our office (415-XXX-XXX) if you develop these symptoms:
  1. Fever of greater than 101.5°F
  2. Nausea or vomiting (especially if you are unable to keep liquids down)
  3. Severe pain at the incision
  4. Pus or foul smelling drainage from the incision (thick, dark yellow drainage)
  5. Persistent diarrhea or more than 10 bowel movements in 24 hours
  6. You are not able to urinate after 8 hours
  7. If you experience dizziness, lightheadedness, or extreme fatigue
  8. Bright red blood from the incision, rectum or ostomy (greater than 1 cup). A small amount of bleeding may be normal, depending on your surgery.

  CALL 911 IF YOU DEVELOP: CHEST PAIN, SUDDEN SHORTNESS OF BREATH, FAINTING AND/OR LOSS OF CONSCIOUSNESS.

  Activity: (unless otherwise instructed)

- No lifting greater than 10 pounds for 4 weeks to avoid developing a hernia at the incision.
- No driving while on narcotic pain medication. Avoid driving for at least the first week after surgery.
- It is normal to feel tired; you may need to take naps or rest during the day.
- You may walk flights of stairs, perform non-strenuous activities, ride in a car and shower
- Continue to walk frequently and increase you stamina!

Diet:

- Small frequent meals may be more easily tolerated after abdominal surgery.   Avoid large meals.
- Drink plenty of fluids. Avoid caffeine, alcohol and fluids with high sugar content because they can cause increased urination, diarrhea, and dehydration. Water, Crystal Light and/or sports drinks (Gatorade) are good choices. Sugar free or low sugar sports drinks are better.

Pain Control:

- Pain from the incision is normal. The pain will vary with activity, but should decrease over time.
- Mild crampy abdominal pain and bloating are normal. This should improve slowly. Eating several small meals instead of a few larger meals will help prevent bloating. Walking before and after meals will also help.
- Pain medication can sometimes cause constipation. Drink plenty of fluids and take stool softeners and/or laxatives as instructed. Stop taking stool softener if you develop loose stools.
- Take your pain medication only as prescribed.
- Narcotic pain medications are best for major pain. Narcotic pain medication can affect your ability to think clearly, drive or operate machinery. Do not drive if you are taking narcotic pain medication. Do not take with alcohol.
- Non-steroidal anti-inflammatory medications (NSAID's), such as ibuprofen, may be prescribed and are good for minor pain and will not alter your ability to think clearly. Common brands are Advil®, Motrin® and Aleve®.
- Acetaminophen - Tylenol® is also an excellent pain reliever for minor pain. Do not take Acetaminophen while taking Percocet® or Norco® or other medications that contain Acetaminophen. Taking more than 4,000mg of Acetaminophen in 24 hours can cause severe liver damage.
- If your pain becomes progressively worse, or if you develop nausea and vomiting call your surgeon's office. 415-XXX-XXXX

Bowel Movements:

- After abdominal surgery your bowel movements (BMs) may not be regular. You may have loose stools or constipation. Your surgeon may send you home with medication or recommendations to help with these problems.

Urination:

- You may experience some mild burning with urination, which will improve in time. If the burning persists, you have difficulty urinating, or you urinate small amounts often, call your surgeon's office. If you went home with a urinary catheter, please make sure that you have a follow up appointment for the catheter to be removed.

- **Sleep:**
- You may find you don’t sleep as well after surgery and being in the hospital. This will get better with time. We do not usually recommend sleeping pills unless you were taking them before surgery.

Incision Care:

- Unless you are told otherwise, you may shower. No tub baths, Jacuzzi or swimming until your surgeon gives the OK.
- The incision does not need to be covered. If there is a small amount of drainage cover the area with gauze to protect your clothing and prevent rubbing.
- A small amount of yellow/red/clear drainage from the incision is normal. If you notice thick, dark yellow drainage or, foul smelling drainage or redness at or around the incision (like a spreading sunburn), please call the surgeon’s office as this may indicate infection, especially if you have a fever over 101˚F.
- Most healing takes place by 6 weeks after surgery. The scar will continue to soften, and the skin will become lighter in color over the next year. Keep your incision covered from sunlight for the first few months, or use sunscreen to protect your newly healed skin from sunburn.

Drain Care:

- You may have been sent home with a drain. Your nurse will teach you how to care for it.
- After the drain has been in place for three days you may shower, and change the dressing. Do not take a tub bath or swim.
- Check the skin around the tube. If the skin becomes red, tender or you notice foul smelling thick drainage from the skin, call your physician. A little redness and a small amount of clear to pink drainage are normal.
  -
  - You will go home with instructions, a medication list and prescriptions. If you have questions please call your surgeon.

  The Colorectal Surgery office number is 415-XXX-XXXX
